# Does the choice of tariff matter? A comparison of EQ-5D-5L utility scores using Chinese, UK, and Japanese tariffs on patients with psoriasis vulgaris in Central South China

**DOI:** 10.1097/MD.0000000000007840

**Published:** 2017-08-25

**Authors:** Yue Zhao, Shun-Ping Li, Liu Liu, Jiang-Lin Zhang, Gang Chen

**Affiliations:** a Department of Dermatology, Heping Hospital, Changzhi Medical College, Changzhi, Shanxi; b School of Health Care Management, Shandong University; c Key Laboratory of Health Economics and Policy Research, NHFPC (Shandong University), Jinan; d Department of Dermatology, Xiangya Hospital, Central South University, Changsha, China; e Centre for Health Economics, Monash Business School, Monash University, Clayton, Australia.

**Keywords:** China, EQ-5D-5L, health state utility, psoriasis vulgaris

## Abstract

There is an increasing trend globally to develop country-specific tariffs that can theoretically better reflect population's preferences on health states for preference-based health-related quality-of-life instruments, also known as multiattribute utility instruments. This study focused on the most recently developed 5-level version of EuroQol-5 Dimension (EQ-5D) questionnaire, 1 of the world's most well-known multiattribute utility instruments, and aimed to empirically explore the agreements and known-group validities of applying the country-specific tariff versus tariffs developed from other countries using a sample of psoriasis vulgaris patients in Mainland China.

A convenience sampling framework was adopted to recruit patients diagnosed with psoriasis vulgaris from Xiangya Hospital, Central South University, China, between May 2014 and February 2015. The 5-level EuroQol-5 dimensions (EQ-5D-5L) utilities were scored by using the Chinese, Japanese, and UK tariffs. Health state utilities were compared using a range of nonparametric test. The intraclass correlation coefficients and Bland–Altman plots were used to examine the agreements among the 3 EQ-5D-5L scores. Health state utility decrements between known groups were investigated using both effect size and a regression analysis.

In all, 350 patients (aged 16 years or older) were recruited. There were significant differences among the 3 national tariff sets. Overall, 3 tariffs showed excellent agreements (intraclass correlation coefficient >0.90); however, the wide limits of agreement from the Bland–Altman plots suggest that these tariffs cannot be used interchangeably. The EQ-5D-5L scores using the Chinese-specific tariff showed the best known-group validity than the other 2 tariffs in this Chinese patient sample. The evidence from this study supports the choice of the country-specific tariff to be used in Mainland China.

## Abstract 2

Supplemental Digital Content is available in the text

## 1 Introduction

Cost-utility analysis (CUA) is 1 of most frequently used methods in health economics evaluation. The health outcome of CUA is often measured by quality-adjusted life years (QALYs), which capture both the quality and quantity of life.^[1]^ The key component of QALYs is the health states utility, which lies on a 0 to 1 (death-full health) scale, with negative utilities referring to health states perceived to be worse than death.^[2]^ The multiattribute utility instrument (MAUI), or the preference-based health-related quality of life (HRQoL) instrument, has been widely used to elicit health state utilities from patients. A MAUI consists of 2 components: a health state descriptive system and a tariff (or called “value set,” “preference weights”), which reflects the preference on different health states of a particular population. Commonly used MAUIs include the EuroQol-5 Dimension (EQ-5D),^[3,4]^ the short-form 6-dimensions (SF-6D) derived from the Short-form 36 (SF-36),^[5]^ and the Health Utilities Index.^[6]^ Currently, the EQ-5D is the most widely used MAUI internationally.^[7]^ It plays an important role in the QoL appraisal and CUA internationally.

The EQ-5D instrument contains a 5-item (5-dimension) descriptive system of HRQoL (mobility, self-care, usual activities, pain/discomfort, and anxiety/depression) and a stand-along visual analog scale (VAS). The classical 3-level EQ-5D (EQ-5D-3L)^[3]^ descriptive system has 3 response levels (no problem, some problems, or severe problems) for each dimension. But the EQ-5D-3L instrument has a couple of key limitations. Firstly, it is less sensitive to the mild health changes.^[8,9]^ Secondly, it can suffer from strong ceiling effects when used in both general population surveys and clinical settings.^[10]^ To improve the limitations above, the new 5-level EQ-5D (EQ-5D-5L) instrument was developed by retaining the original 5 dimensions and expanding the number of levels of severity in each dimension from 3 to 5 levels: no problems, slight problems, moderate problems, severe problems, and unable to/extreme problems.^[4]^ Accordingly, 3125 (5^5^) unique health states can be described by the EQ-5D-5L.^[4]^

Currently, there is an increasing trend to develop country-specific tariffs so as to better reflect population's preferences on different health states. When country-specific tariffs are unavailable, researchers usually opt to use the UK tariff,^[11]^ or the tariff derived from a country that was geographically or culturally closer. However, little do we know how large the difference could be in such substitutions. This study aims to empirically investigate whether it matters if we adopt different country-specific tariffs in a clinical setting in mainland China. More specifically, this study examined the agreements and known-group validities of Chinese^[12]^, Japanese,^[13]^and UK tariffs^[11]^ on estimating health state utility scores of a sample of Chinese patients with psoriasis vulgaris. Before the Chinese EQ-5D-5L tariff was established, the UK tariff was usually used in China. The Japanese tariff was also compared in this study, considering the geographical proximity and similar cultural backgrounds between Japan and China, which would make the comparison valuable. To our knowledge, this is the first study to compare the differences of EQ-5D-5L utility scores using the Chinese, UK, and Japanese tariffs based on the Chinese psoriasis vulgaris patients. The findings from the study will provide valuable suggestions to the choice of country-specific tariff in health states valuation.

## 2 Methods

### 2.1 Study sample

Psoriasis vulgaris is a chronic inflammatory skin disease, characterized by the presence of scaly plaques and papules.^[14]^ There are serious systematic complications, such as arthritis, hypertension, and atherosclerosis, exerting significant negative impact on the QoL of psoriasis patients.^[15]^ In China, the prevalence of psoriasis is 0.47%.^[16]^

Participants who were diagnosed with psoriasis vulgaris were recruited successively from the outpatient clinics of Xiangya Hospital, Central South University, China, between May 2014 and February 2015. Patients were excluded if they were unwilling to give an informed consent; unable to understand the questionnaire; or the patient was younger than 16 years at the time of the survey. Face-to-face interviews were performed by the 2 investigators (Y.Z., J.L.Z.). The face-to-face interviews included sociodemographic information, and generic and disease-specific QoL. Clinical information was also collected from the hospital information system. Informed consent was obtained from all respondents before the interviews.

### 2.2 Self-assessed QoL instruments

#### 2.2.1 Psoriasis Disability Index

The Psoriasis Disability Index (PDI) focuses on the functional lifestyle disabilities caused by psoriasis.^[17]^ The PDI includes 15 items which can be grouped into 5 subscales: daily activities, work, personal relations, leisure, and treatment. All items are rated on a 4-point scale, with responses of “not at all,” “a little,” “a lot,” and “very much,” scored as 0, 1, 2, and 3, respectively. A summary score (range 0–45) can be generated by adding all 15 items together. A higher PDI score means greater limitations in life because of psoriasis. The validated Chinese version PDI was adopted in this study.^[18]^

#### 2.2.2 EQ-5D-5L country-specific tariffs

The Chinese EQ-5D-5L tariff was elicited from the general urban population living in 5 cities of China^[12]^. A total of 86 health states were valued using the composite time trade-off (TTO) technique. The Japanese^[13]^ and UK^[11]^ EQ-5D-5L tariffs were both elicited from general populations in each country, combining a composite TTO technique (on 86 health states) and a discrete choice experiment (DCE) (on 196 pairs of health states). A hybrid modeling approach was used to combine both TTO and DCE data for estimating the final tariff. Three national EQ-5D-5L tariffs are presented in the Supplementary Table 1.

### 2.3 Physician-assessed index: Psoriasis Area and Severity Index

The Psoriasis Area and Severity Index (PASI) is commonly used by the physician to assess both intensity and extent of the psoriatic plaques separately for head, trunk, upper, and lower extremities. The PASI score ranges from 0 (no psoriasis) to 72 (very severe psoriasis). The PASI score could be grouped into 3 severity levels of psoriasis: PASI <7 (mild severity), PASI 7 to 12 (moderate severity), and PASI >12 (severe severity).^[19]^

### 2.4 Data analysis

The EQ-5D-5L utility scores were calculated using the Chinese, Japanese, and UK tariffs separately. The differences among the 3 utility scores were compared using the Friedman test and the Wilcoxon signed-rank tests. Nonparametric tests were chosen because the normality assumption required for parametric tests was not satisfied by our sample. The correlations between 3 EQ-5D-5L utility scores and EuroQol-visual analog scale (EQ-VAS) scores, PASI scores, and PDI scores were examined by Spearman rho correlation coefficients. The agreements among the 3 national tariffs were assessed employing intraclass correlation coefficients (ICCs)^[20]^ and Bland–Altman plots.^[21]^ Absolute agreement was considered strong for ICC values higher than 0.70.^[20]^ The differences in utility scores between different psoriasis vulgaris severity groups (known groups) were studied by using the Cohen effect size (the mean differences divided by the standard deviation [SD] of the less severity group).^[22]^ Known-groups validity was further studied using a regression analysis. It is hypothesized that psoriasis patients at a more severity stage according to the PASI scores should have a significantly lower mean EQ-5D-5L utility score. Owing to a large proportion of patients were classified as in full health (ie, health state utility score equals to 1 according to the EQ-5D-5L descriptive system), a Tobit model was used in the regression analysis instead of the classical ordinary least squares model.^[23]^ With the exception of the Bland-Altman plot, which was performed using MedCalc version 16.8 (MedCalc Software, Ostend, Belgium), all other statistical analyses were conducted using Stata version 12.1 (StataCorp LP, College Station, TX).

### 2.5 Ethics approval and consent to participate

The study was approved by the Ethics Review Board of the School of Medicine, Shandong University (Reference No. LL-201401044).

## 3 Results

### 3.1 Characteristics of participants

Three hundred fifty patients who were diagnosed with psoriasis vulgaris were invited to participate in this study, and all patients consented to be interviewed. The sociodemographic and clinical characteristics of the sample are shown in Table 1. Patients’ median age was 39 years (range 16–80 years), and 69.7% were male, 88.6% of patients received junior high or above education, and 59.7% lived in the city. The median family income was ¥40,000 per year. The mean duration of psoriasis was 8.4 years (SD 9.34; range 0.1–48), with 57.3%/24.6%/18.1% of participants were classified as mild/moderate/severe psoriasis. The mean of EQ-VAS scores was 72.79 (SD 15.65; range 10–100) and the mean of PDI scores was 8.59 (SD 6.26; range 0–30).

**Table 1:**
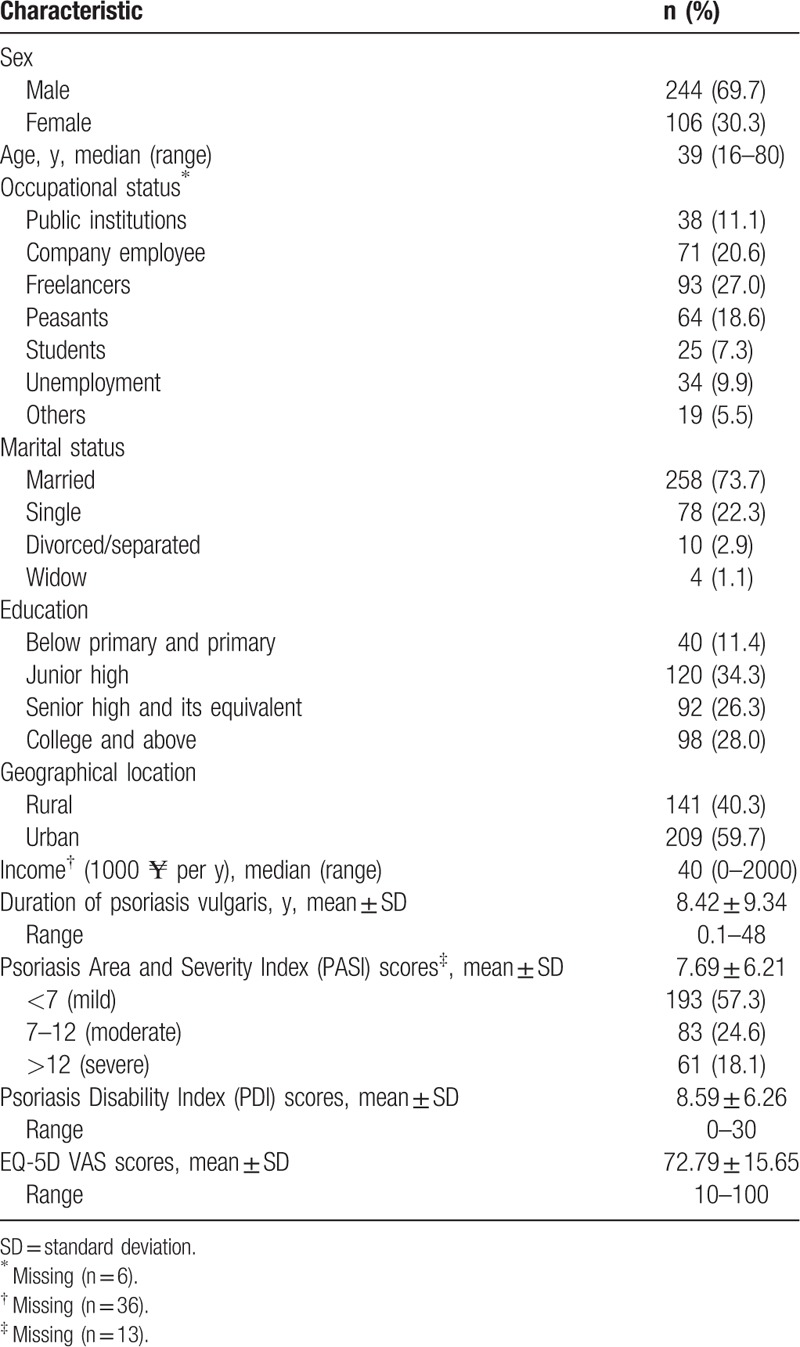
Sociodemographic and clinical characteristics of psoriasis vulgaris patients (n = 350).

### 3.2 Comparisons on EQ-5D-5L utility scores derived from 3 country-specific tariffs

**Figure 1:**
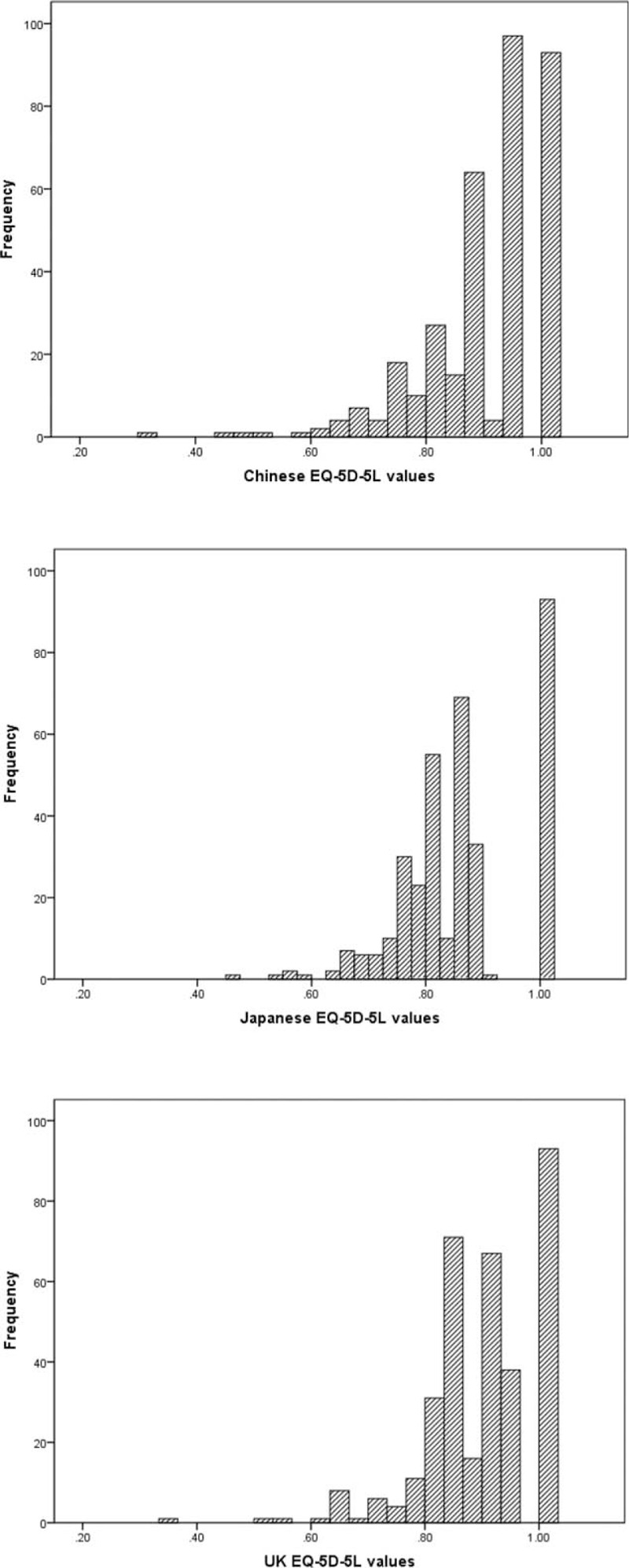
Distribution of EQ-5D-5L scores from 3 tariffs among psoriasis vulgaris patients. EQ-5D-5L = 5-level EuroQol-5 dimensions.

All 3 EQ-5D-5L utility scores were left-skewed distributions (Fig. 1). The mean ± SD (median) of EQ-5D-5L utility scores derived from Chinese, Japanese, and UK tariffs were 0.902 ± 0.104 (0.942), 0.862 ± 0.103 (0.860), and 0.901 ± 0.093 (0.924), respectively (Table 2, panel A). There were statistically differences among EQ-5D-5L utility scores derived from 3 country-specific tariffs (Friedman test, *χ*^2^ = 271.99, *P* < .001). The differences in mean utility scores were larger between Chinese and Japanese tariffs (difference 0.0394, 95% confidence interval [CI] 0.035–0.044), Japanese and UK tariffs (difference −0.0387, 95% CI −0.043 to −0.035) than between Chinese and UK tariffs (difference 0.001, 95% CI −0.003 to 0.005).

**Table 2:**
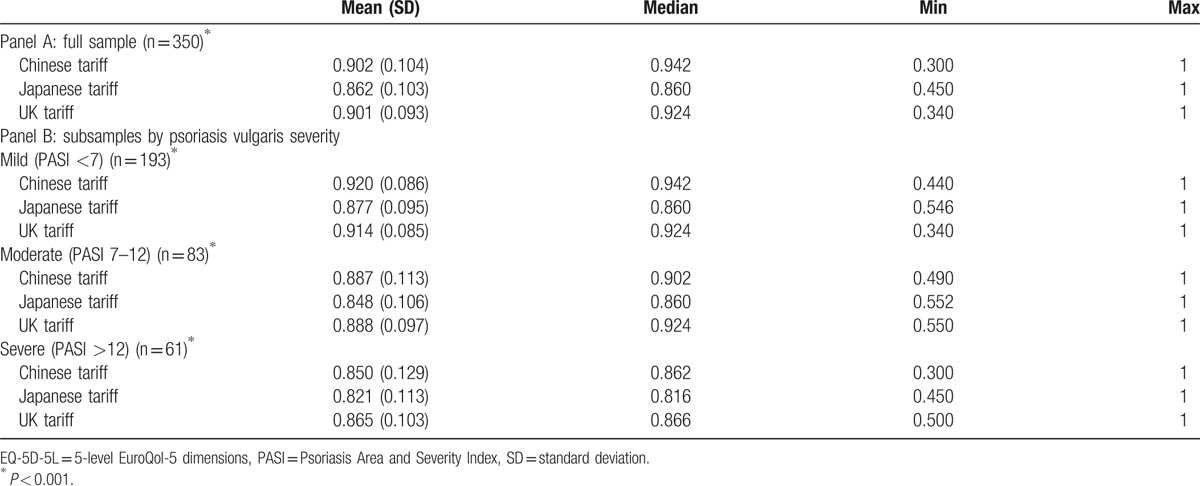
EQ-5D-5L utility scores of patients based on three country-specific tariffs.

Further analyses were conducted by comparing the 3 EQ-5D-5L utility scores according to the severity levels of psoriasis vulgaris (Table 2, panel B). There exist significant differences among the 3 utility scores within each severity level. The pair-wise comparisons of utility scores found significant difference on all pairs (*P* < .001), except for the comparison between Chinese and UK tariffs among patients with moderate (*P* = .232) or severe psoriasis (*P* = .114).

Table 3 shows that regardless of which tariff was used, the EQ-5D utility scores were significantly associated with both self-assessed EQ-VAS and PDI scores, and also physician-assessed PASI scores. Among them, the largest magnitudes of correlations were found between Chinese tariff-based EQ-5D-5L and EQ-VAS/PASI, and between Japanese tariff-based EQ-5D-5L and PDI.

**Table 3:**
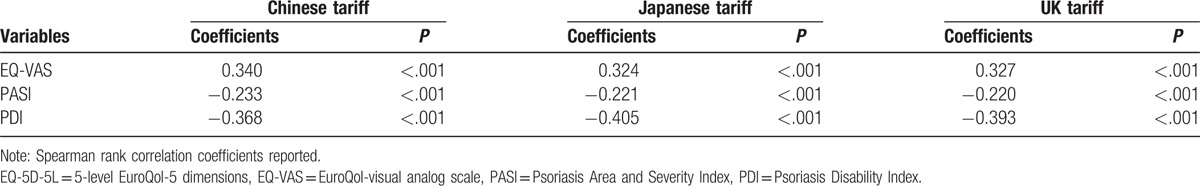
Correlations between 3 EQ-5D-5L utilities and other quality-of-life scores.

### 3.3 Agreements of 3 country-specific tariffs

**Figure 2:**
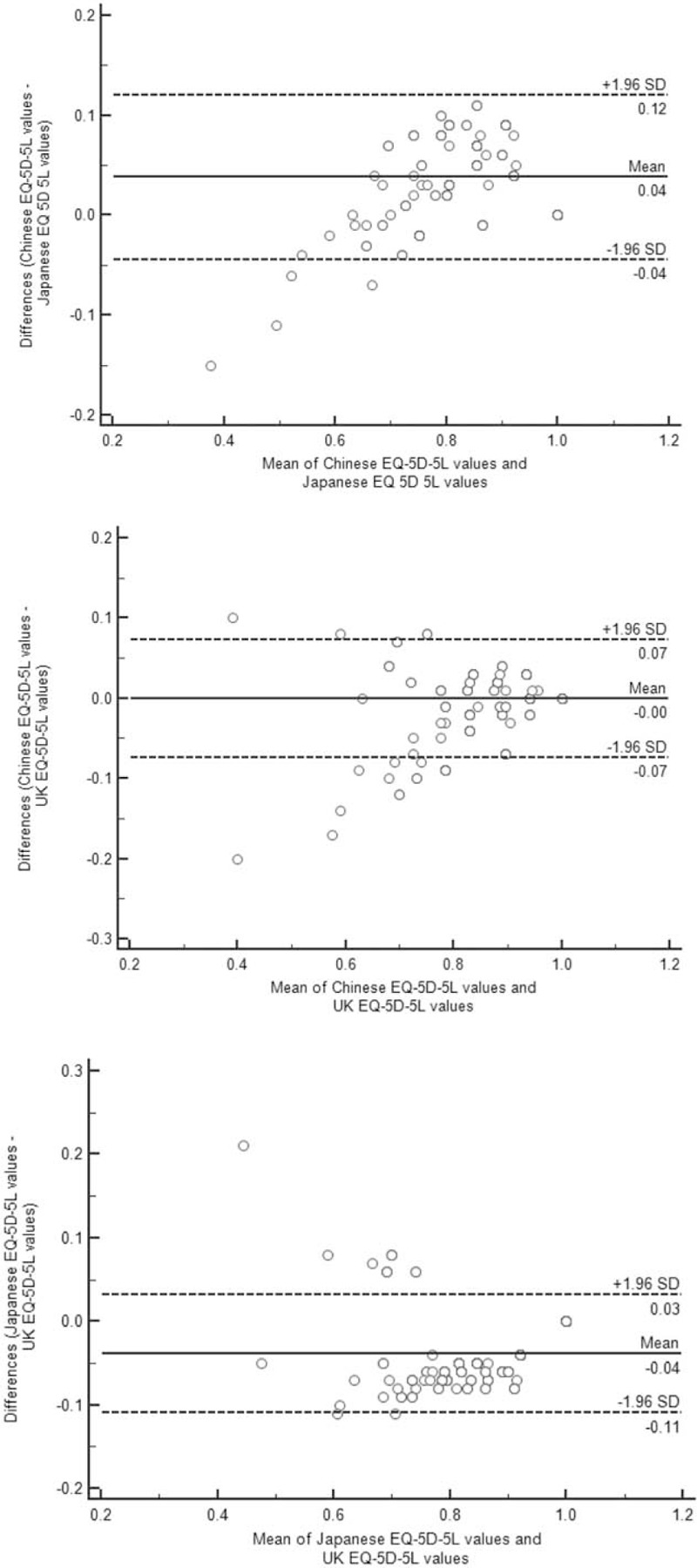
The Bland–Altman plots of EQ-5D-5L scores derived from 3 tariffs. EQ-5D-5L = 5-level EuroQol-5 dimensions.

The overall ICC among three EQ-5D-5L utility scores was 0.957 (95% CI 0.889–0.978), indicating an excellent agreement among 3 tariffs. Pair-wise ICCs support the above conclusion that ICC = 0.922 (95% CI 0.600–0.970), 0.964 (95% CI 0.956–0.971) between Chinese and Japanese/UK tariffs, and 0.928 (95% CI 0.495–0.974) between Japanese and UK tariffs. Figure 2 shows the Bland–Altman plots between each pair of EQ-5D-5L scores. The mean of the differences and the limits of agreement (LOA) are indicated by lines. It can be seen that the range of LOAs ranged between 0.14 and 0.16.

### 3.4 Known-groups validities

Table 4 presents the effect sizes between each of the 2 severity levels. It can be seen that the absolute values of effect sizes were consistently the highest when EQ-5D-5L scored using the Chinese tariff. The evidence was mixed regarding whether the Japanese or UK tariff produced the second highest effect sizes among 3 pairs of comparisons.

**Table 4:**
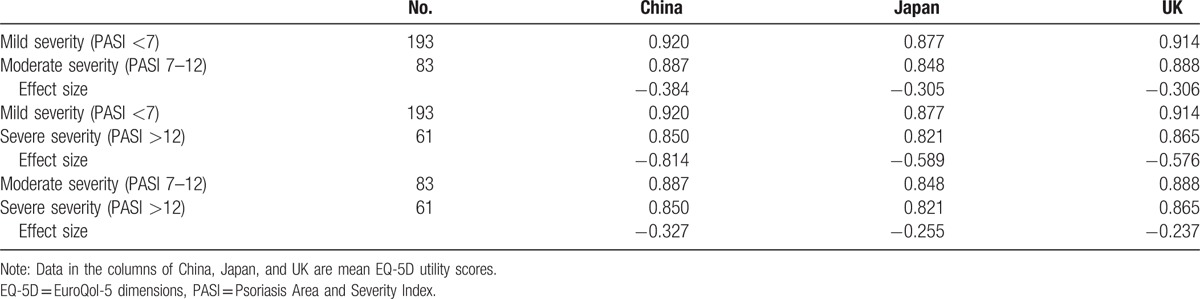
Known-groups validity of the EQ-5D index scores using country-specific tariffs in different psoriasis vulgaris severities.

Table 5 further reports the regression analysis results. Controlling for the confounding factors, along with an increased severity of psoriasis, EQ-5D-5L utility scores decreased significantly regardless of which tariff was used. Compared with the mild severity, the magnitudes of decrements on EQ-5D-5L were the largest when EQ-5D-5L was scored using the Chinese tariff, followed by Japanese and UK tariffs. Other 2 significant variables included in the regression were disease duration and sex. On average, an additional year of disease duration was found to be associated with a 0.002 utility increase. In addition, males had significant higher utility score than females.

**Table 5:**
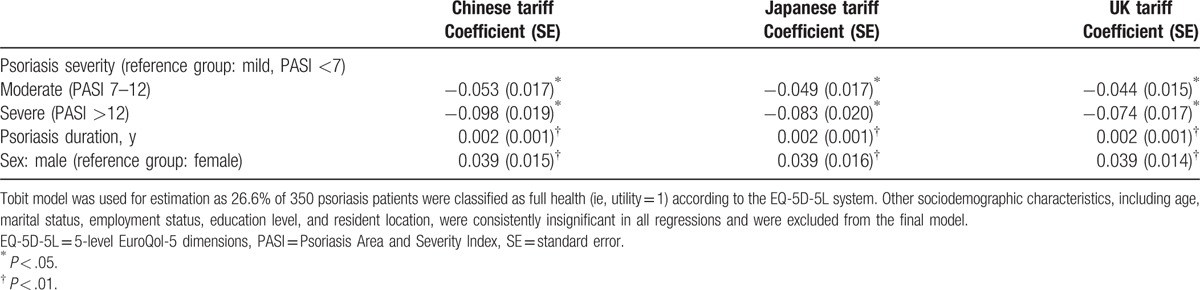
Known-groups validity of 3 EQ-5D-5L national tariffs.

## 4 Discussion

There is an increasing interest to develop country-specific tariffs that can theoretically better reflect population's preferences on health states in conducting CUA. Based on psoriasis patients in Central South China, this study empirically investigated the correlations, agreements, and sensitivity of 3 EQ-5D-5L country-specific tariffs.

Results from this study firstly show that mean EQ-5D-5L utility scores varied according to the different country-specific tariffs been applied. The mean utility score was higher based on the Chinese tariff than the UK/Japanese tariff. This observation is consistent with the previous study conducted in the Chinese general population using the EQ-5D-3L tariffs.^[24]^ However, the absolute mean utility differences between each of the 2 tariffs varied in these 2 Chinese studies. Regarding the UK versus Japanese tariff, this study found a significantly higher mean utility score from the UK tariff than the Japanese tariff. An observation differs from a previous study conducted in Thailand using EQ-5D-3L among patients with type 2 diabetes^[25]^ (which showed statistically insignificant difference). The discrepancy observed above may be attributable to the difference in sample populations and/or different versions of EQ-5D instruments (and corresponding tariffs) been used.

Secondly, an excellent absolute agreement among 3 country-specific tariffs was demonstrated according to the ICCs (ie, all ICC > 0.9). However, the smallest LOA (0.14) from the Bland–Altman plots was still wider than the minimally important difference (MID) of around 0.069, based on the Chinese EQ-5D-5L tariff.^[26]^ This suggests that none of the 3 tariffs could be regarded as interchangeable. The relatively higher agreement between utility scores derived from Chinese and UK tariffs may owe to the similar function form (ie, no constant terms) that was adopted when deriving Chinese and UK tariffs.^[11,12]^

Thirdly, the Chinese-specific EQ-5D-5L tariff was found to be the most sensitive among psoriasis vulgaris patients in China. The EQ-5D-5L utility scores calculated from all 3 tariffs were significantly associated with HRQoL measured by PASI, PDI, and EQ-VAS scores. It should be noted that the Chinese tariff had the best known-groups validity among this psoriasis vulgaris Chinese patient sample according to both the effect size, and the magnitudes of mean utility decrements from mild to more severe disease status (after controlling for other confounding factors). With regard to the Japanese and UK tariffs, the results were inconclusive. This empirical evidence supports the value of developing of a country-specific tariff.

This study has 3 caveats. Firstly, psoriasis vulgaris patients were recruited from 1 tertiary hospital located in Central South China. However, there is no evidence to suggest that psoriasis vulgaris patients from different regions of China have substantial different reporting patterns on how psoriasis vulgaris impacts on their HRQoL. The regression analysis results further suggest that only severity, disease duration, and sex had significant impact on psoriasis vulgaris patients’ HRQoL. Other potential co-founding factors, such as geographic location (ie, whether patients come from urban or rural regions), occupation, education levels, and age were all insignificant. Secondly, the key conclusion from this study that the Chinese-specific EQ-5D-5L tariff is indeed the most suitable one to use for Chinese population may not be generalized to other patient populations and/or other countries. As country-specific EQ-5D-5L tariffs have just been published, it is expected that more evidence from other countries and/or other diseases may be reported in the near future. Thirdly, only a cross-sectional survey was carried out and as such we will not be able to test the responsiveness of different EQ-5D-5L tariffs. Despite above limitations, this is 1 of the first studies internationally to empirically investigate whether the choice of county-specific EQ-5D-5L tariffs matters in practice.

## 5 Conclusions

There were excellent agreements among the Chinese, Japanese, and UK country-specific EQ-5D-5L tariffs; however, 3 tariffs cannot be used interchangeably. The Chinese EQ-5D-5L tariff demonstrated the best known-groups validity among psoriasis vulgaris patients with different severities in China. The evidence from this study supports to use the country-specific tariff upon availability.

## Supplementary Material

Supplemental Digital Content
