# Supplementary material for: Does the choice of tariff matter? A comparison of EQ-5D-5L utility scores using Chinese, UK, and Japanese tariffs on patients with psoriasis vulgaris in Central South China
Source: Medicine (Baltimore). 2017 Aug 25;96(34):e7840. doi: 10.1097/MD.0000000000007840 (PMC5572015; doi:10.1097/MD.0000000000007840)
Supplement: Supplemental Digital Content [file medi-96-e7840-s001.docx]

**Supplementary Table 1 Chinese, Japanese, and UK tariffs for the EQ-5D-5L utilities calculation**

| Variables | Definition | UK model | China model | Japan model |
| --- | --- | --- | --- | --- |
| Constant | At least one level at 2, 3, 4 or 5 | NA | NA | 0.062 |
| Mobility |  |  |  |  |
| Level 2 | Mobility at level 2 | 0.049 | 0.066 | 0.065 |
| Level 3 | Mobility at level 3 | 0.061 | 0.158 | 0.113 |
| Level 4 | Mobility at level 4 | 0.205 | 0.287 | 0.179 |
| Level 5 | Mobility at level 5 | 0.266 | 0.345 | 0.240 |
| Self-Care |  |  |  |  |
| Level 2 | SC at level 2 | 0.055 | 0.048 | 0.038 |
| Level 3 | SC at level 3 | 0.074 | 0.116 | 0.070 |
| Level 4 | SC at level 4 | 0.175 | 0.210 | 0.118 |
| Level 5 | SC at level 5 | 0.210 | 0.253 | 0.161 |
| Usual Activities |  |  |  |  |
| Level 2 | UA at level 2 | 0.049 | 0.045 | 0.057 |
| Level 3 | UA at level 3 | 0.065 | 0.107 | 0.092 |
| Level 4 | UA at level 4 | 0.168 | 0.194 | 0.155 |
| Level 5 | UA at level 5 | 0.184 | 0.233 | 0.173 |
| Pain/Discomfort |  |  |  |  |
| Level 2 | P/D at level 2 | 0.058 | 0.058 | 0.041 |
| Level 3 | P/D at level 3 | 0.073 | 0.138 | 0.068 |
| Level 4 | P/D at level 4 | 0.267 | 0.252 | 0.124 |
| Level 5 | P/D at level 5 | 0.330 | 0.302 | 0.193 |
| Anxiety/Depression |  |  |  |  |
| Level 2 | A/D at level 2 | 0.076 | 0.049 | 0.078 |
| Level 3 | A/D at level 3 | 0.101 | 0.118 | 0.111 |
| Level 4 | A/D at level 4 | 0.286 | 0.215 | 0.173 |
| Level 5 | A/D at level 5 | 0.291 | 0.258 | 0.197 |

Note: The constant term is a number which intercept associated with any move away from full health (‘‘11111’’).

Abbreviations: NA, not applicable; SC, self-care; UA, usual activities. A/D, anxiety/depression; P/D, pain/discomfort.
